# A Novel 7-Methylguanosine (m7G)-Related Gene Signature for Overall Survival Prediction in Patient with Clear Cell Renal Cell Carcinoma

**DOI:** 10.1155/2023/9645038

**Published:** 2023-04-08

**Authors:** Yongxin Fu, Jiawu Wang, Zhiya Hu, Yang Gou, Yisen Li, Qing Jiang

**Affiliations:** Department of Urology, The Second Affiliated Hospital of Chongqing Medical University, Chongqing, China

## Abstract

Clear cell renal cell carcinoma (ccRCC) is the most common pathology type of renal cancer that has an abysmal prognosis. Although a crucial role for 7-methylguanosine modification in cancer cell development has been reported, its role in ccRCC remains uncertain. This study was conducted to determine the efficacy of predictive biomarkers based on m7G-related genes in ccRCC. Firstly, we extracted clinical data and gene expression profiles of ccRCC patients from publicly accessible databases. It identified that 22 of the m7G-related 34 genes were related to overall survival, and 5 of the 22 genes were significantly expressed differently in tumor tissues. Based on Lasso regression analysis, five optimal genes (CYFIP2, EIF4A1, NUDT1, NUDT10, and NUDT4) were chosen to build a new predictive risk model in the TCGA cohort. Validation was carried out with the E-MTAB-1980 cohort. Then, a prognostic nomogram was erected, including the m7G-related gene risk score, age, histological grade, and stage status. Further studies and analysis showed that immune cell infiltration might be associated with the m7G-related risk genes. In addition, the relationship between gene expression and drug response was evaluated by the Pearson correlation test. Therefore, the risk signature with five selected m7G-related genes may be a promising prognostic biomarker and contribute to standardized prognostic assessment for ccRCC.

## 1. Introduction

Ninety percent of all renal malignancies are renal cell carcinomas (RCC), a prevalent malignancy in the urinary system [1]. ccRCC, the most common subtype of RCC, accounts for approximately 70% of RCC and is also one of the most aggressive subtypes [2]. The early symptoms of renal clear cell carcinoma are not obvious, with only 6–10% of patients presenting with typical symptoms such as backache, an abdominal mass, or hematuria [3]. More than about 30% of ccRCC patients already have metastasis or local progression at the first diagnosis [4], so it is necessary to diagnose ccRCC at an early stage. Although surgery is the best therapy method for ccRCC, nearly 30% of patients develop local recurrence and metastasis after surgically removing local ccRCC [5]. Despite the advent of targeted drugs and immune checkpoint inhibitors, patients with metastatic renal cancer still have a low overall survival rate. Hence, it is crucial to discover effective and novel biomarkers to provide novel molecular targets for adjuvant therapies and improve patient outcomes.

In the last decade, the role of RNAs in cellular processes has attracted more and more attention. Over 150 types of RNA modifications have been identified to date [6, 7], including N7-methylguanosine, N1-methyladenosine,2′-O-methylation, N6-methyladenosine, and 5-methylcytosine [8–10]. In general, M7G, an RNA modification with a positive charge [11], is present in the eukaryotic mRNA, tRNA, rRNA, and microRNAs [12], which is required for nearly all phages that participate in the expression of mRNAs, such as transcription elongation [13], pre-mRNA splicing [14], polyadenylation [15], nuclear export [16], and translation [17]. Although some literature reported that m7G modification is linked to the development of various cancers, including lung cancer [18] and colon cancer [19], the relationship between m7G RNA methylation regulators and ccRcc remains widely unclear.

We first downloaded mRNA expression and clinical data from public databases. Then, selected five optimal genes and constructed a predictive model. The model's predictive value has been confirmed via various survival analyses. Moreover, we also validated the accuracy of the model in the E-MTAB-1980 cohorts. Furthermore, we set up a nomogram to enhance individualized prognosis assessment. Finally, we elucidate the underlying mechanism of ccRCC through functional analysis and explore the relationship between risk genes and chemosensitivity.

## 2. Materials and Methods

### 2.1. Data Acquisition

**Figure 1:**
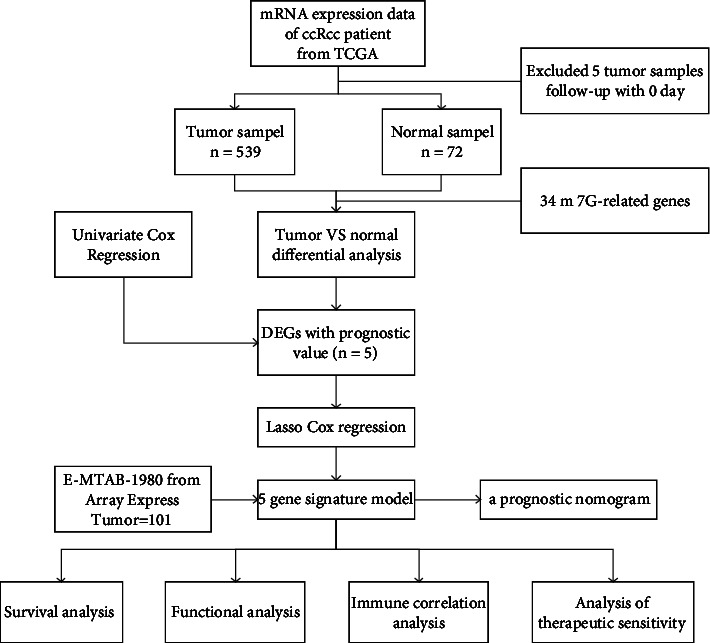
The overall workflow of this study.

We acquired the RNA sequence data of 539 tumors and 72 normal tissues of ccRCC and clinical information (*n* = 537) in the TCGA database. In addition, 101 patients in the E-MTAB-1980 cohort with clinicopathological information and RNA sequence data were also downloaded from the ArrayExpress database. Reference [20]. Our analysis excluded patients who had no follow-up days during the survival period. Thirty-four m7G-related genes were obtained from earlier studies [21], and gene sets: gomf-m7G-5-pppn-diphosphatase-activity, gomf-RNA-7-methylguanosine-cap-binding, and gomf-RNA-cap-binding (https://www.gsea-msigdb.org/gsea/index.jsp). Basic clinical information is summarized in Table 1. A flow chart of our overall study is displayed in Figure 1.

### 2.2. Prognostic Model Construction and Validation

Differentially expressed genes (DEGs) in ccRCC normal samples and tumor samples in the TCGA cohort were processed using the “Limma” R package, and the filter conditions were set (fdrFilter <0.05, logFCfilter >1). Then, a univariate Cox analysis of survival outcomes was performed to screen the genes with prognostic values. The “Venn” R package was used to select m7G-related DEGs with predictive values through DEGs and prognostically valuable genes. Furthermore, we used the “pheatmap” package to display heatmaps and the “Survival” package to show forest maps to represent differences between groups. In the TCGA cohort, the “Survival” and the “glmnet” packages were applied for prognostic risk characteristics. The risk score of each patient was estimated as follows: Risk score = ∑ (gene expression × corresponding coefficient. Then, we calculated the risk score of each patient according to the above formula and divided all patients into high-risk and low-risk groups with the median score. “Survminer” and “Survival” of the R language were introduced to evaluate overall survival (OS) based on the Kaplan–Meier (K–M) method. The “timeRoc” of the R package was applied to assess the accuracy of the related gene model. The “pheatmap” of the R package was used to describe the risk scores and corresponding survival times. The R packages “Rtsne” and “GGplot2” were used to investigate whether there was a difference in distribution between the two groups of at-risk patients. We further processed the same analyses to validate the model's predictive performance in the E-MTAB-1980 cohorts.

### 2.3. The Construction of a Predictive Nomogram Using Risk Scores and Clinical Factors

We performed the analysis of univariate and multivariate Cox regression further to examine the relationship between OS and clinical factors. Then, establishing a nomogram including a risk score and the independent prognostic clinical characteristics, we can assess the probability of survival at 1, 3, and 5 years for ccRCC patients. In order to measure this nomogram's predictive precision, calibrate curve analysis was used.

### 2.4. Functional and Immune Infiltration Enrichment Analyses

Using the package ClusterProfiler, we used GO and KEGG to analyze risk-related DEGs. The ESTIMATE algorithm assessed the association between risk score and stromal score or immune score. “GSVA” and “GSEABASE” were utilized to assess immune functions and cells among risk-related DEGs.

### 2.5. Drug Response Analysis

We obtained the NCI-60 data from the CellMiner platform and used the Pearson correlation test to assess the relation of m7G-related gene expressions with drug response.

### 2.6. Statistics Analysis

Statistical analyses were conducted using the R software (version 4.1.0) and the PERL programming language (version 5.32.1). Statistical significance is set at *P* < 0.05, and *P* values follow a double-tailed distribution. To calculate the adjusted *P* value, Benjamini-Hochberg was invoked.

## 3. Results

### 3.1. Differential Expression of MRGs

**Figure 2:**
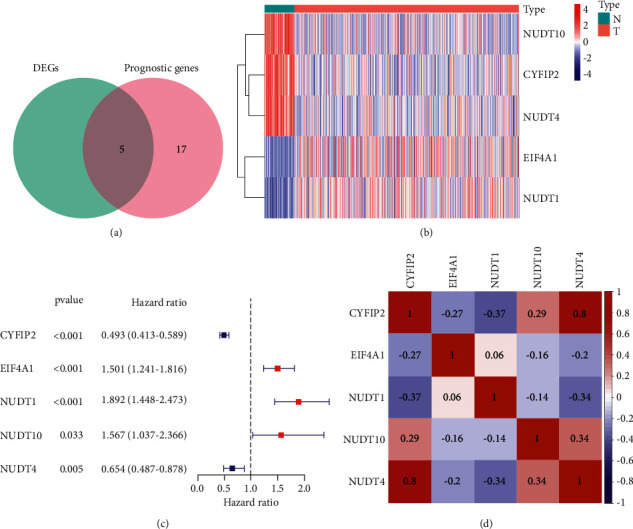
Intersection of differentially expressed m7G-related genes and survival-associated m7G-related genes. (a) Venn diagram showing the five m7G-related genes associated with survival between differentially expressed genes and prognostic genes. The green circle represents differentially expressed genes, and the pink circle represents prognostic genes. (b) Heatmap showing the five m7G-related associated with survival. The red rectangle represents tumor tissue, and the blue rectangle represents normal tissue. (c) The forest figure of the 5 key m7G-related genes in univariate Cox regression. The blue line represents a 95% confidence interval. The position of the square represents the hazard ratio. (d) The correlation network of 5 m7G-related genes, in which different colors represent the correlation coefficients. The red represents a positive relationship, and the blue represents a negative relationship.

Most of the m7G-related expressed genes (5/34, 14.7%) were differentially expressed between nontumorous tissues and tumorous tissues in the TCGA cohort (adjusted *P* < 0.05, logFCfilter >1). We screened out 22 genes associated with OS (*P* < 0.01) via univariate Cox regression analysis. Thus, five m7G-related genes were identified, as indicated by the Venn diagram (Figure 2(a)). Afterward, the heatmap demonstrated the expression levels in normal and tumor samples (Figure 2(b)). Furthermore, we calculated the *P* values, hazard ratios, and 95% confidence intervals (CI) of each gene displayed in forest plots (Figure 2(c)). Finally, we visualized five prognostic m7G-related DEG interactions with correlation networks (Figure 2(d)).

### 3.2. Generation of the Predictive Risk Model and Survival Analyses in the TCGA Database

**Figure 3:**
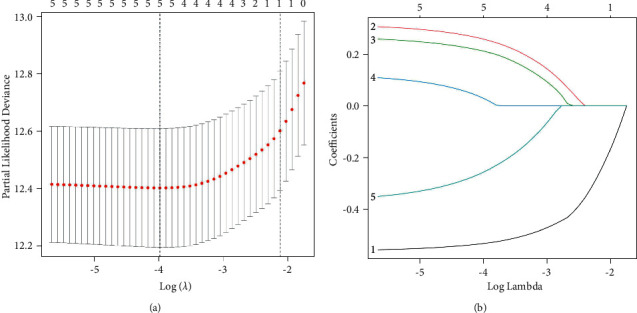
Identification of five optimal MRGs. (a) Partial likelihood deviance was plotted against log (*λ*). The vertical dotted lines indicate the *λ* value with minimum error. The largest *λ* value is where the deviation is within one standard error (SE) of the minimum. (b) Least absolute shrinkage and selection operator (Lasso) coefficient profiles of MRGs in ccRCC.

**Figure 4:**
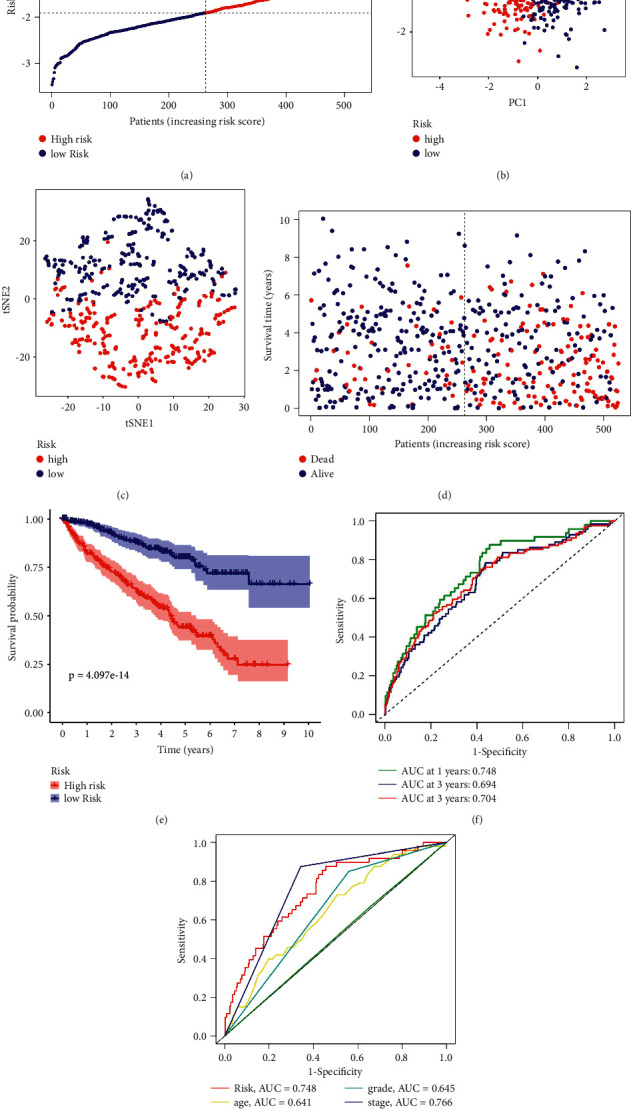
Survival analyses of the 5-gene model in the TCGA cohort. (a) The distribution of the risk scores in the TCGA cohort. (b) Principal component analysis plots display the established gene signature expression distribution in different risk groups. (c) *t*-Distributed stochastic neighbor embedding plots reveal the patients' distribution in different risk groups. (d) The distributions of the risk scores and corresponding survival times of all patients in the TCGA cohort. (e) OS-based K-M survival curves for the patients in the high and low-risk groups in the TCGA cohort. (f) AUC of time-dependent ROC curves verified the prognostic performance of the risk score in the TCGA cohort. (g) AUC of clinical ROC curves verified the prognostic performance of the risk score in the TCGA cohort.

By lasso regression analysis, we discovered that five m7G-related genes fit the model (Figure 3(a) and 3(b)). Among them, EIF4A1, NUDT1, and NUDT10 are high-risk genes, while CYFIP2 and NUDT4 are assigned to low-risk genes. In order to establish the prognosis model based on the expression levels of the selected genes, we computed risk scores using the following formula: risk score = (−0.532106574444019^*∗*^CYFIP2) + (0.257284742647963^*∗*^EIF4A1) + (0.210867606644546^*∗*^ NUDT1) + (0.0273145419183592^*∗*^NUDT10) + (−0.254461172952311^*∗*^NUDT4). Five m7G-related genes model was identified in the TCGA cohort. We categorized all patients based on the median risk scores into two groups: high-risk (*n* = 262) and low-risk (*n* = 263). (Figure 4(a)). The results of PCA and *t*-SNE analysis of MRGs indicated that different risk groups of patients were entirely distributed differently (Figures 4(b) and 4(c)). Furthermore, we noticed that the high-risk patients died more frequently than the low-risk patients when we took survival outcomes into consideration (Figure 4(d)). Therefore, the predictive capacity for the clinical prognosis of the model was evaluated using the K-M survival and time-dependent ROC analyses, respectively. The results are as follows: survival rates for patients assigned to the high-risk group were significantly worse than those in the low-risk group according to the K-M survival curve (*P*=4.097*e* − 14) (Figure 4(e)). During 1 year, 0.748 AUC was recorded, followed by 0.694 at 2 years, and 0.704 at 3 years (Figure 4(f)) and reached 0.748 at risk, 0.766 at stage, 0.645 at grade, 0.641 at age, and 0.506 at gender in the ROC analysis (Figure 4(g)).

### 3.3. Survival Analyses of the E-MTAB-1980 Cohorts for Verification

**Figure 5:**
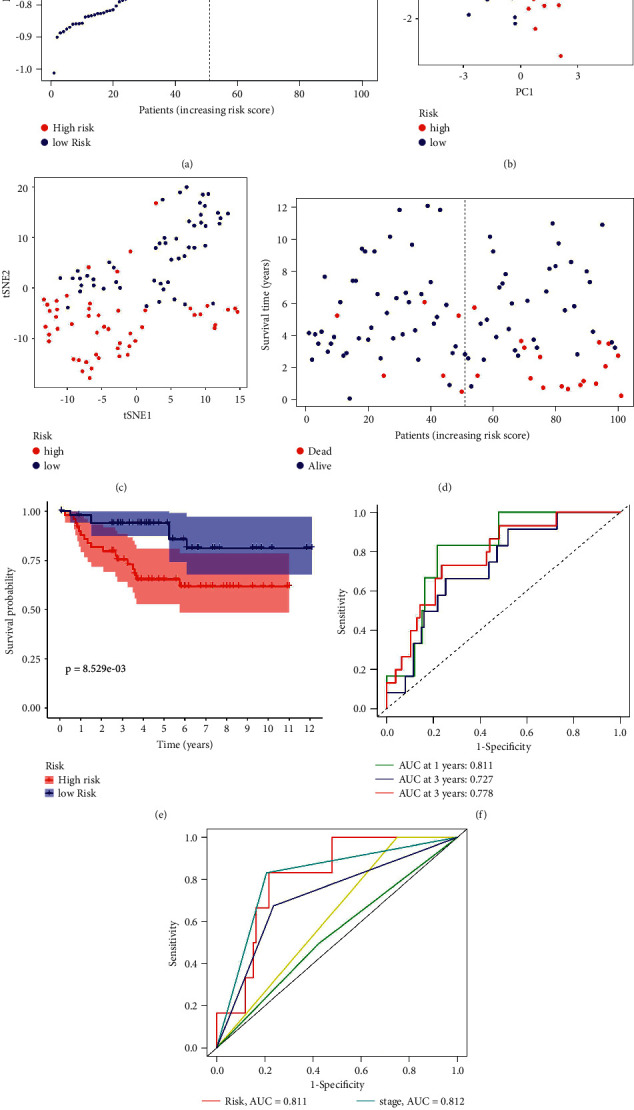
Validation of the 5-gene model in the E-MTAB-1980 cohort. (a) The distribution of the risk scores in the E-MTAB-1980 cohort. (b) Principal component analysis plots display the established gene signature expression distribution in different risk groups. (c) *t*-Distributed stochastic neighbor embedding plots reveal the patients' distribution in different risk groups. (d) The distributions of the risk scores and corresponding survival times of all patients in the E-MTAB-1980 cohort. (e) OS-based K-M survival curves for the patients in the high- and low-risk groups in the E-MTAB-1980 Cohort. (f) AUC of time-dependent ROC curves verified the prognostic performance of the risk score in the E-MTAB-1980 cohort. (g) AUC of clinical ROC curves verified the prognostic performance of the risk score in the E-MTAB-1980 cohort.

We also calculated the risk score for the E-MTAB-1980 cohorts for validation (*n* = 101). In light of the median risk score, patients were divided into two sets: those at high-risk (*n* = 50) and those at low-risk (*n* = 51). (Figure 5(a)). MRGs were reduced in dimension by PCA and T-SNE analysis, and then we found that different risk groups were distributed in two different directions (Figures 5(b) and 5(c)). Additionally, the death rates in the high-risk group were higher than those in the low-risk group. (Figure 5(d)). In addition, we performed analyses of K-M survival and time-dependent ROC curves, which suggested that both were significant in assessing the prognostic value of the risk score model. Survival curves showed that patients in the high-risk group had significantly worse OS than patients in the low-risk group (*P*=1.53*e* − 03) (Figure 5(e)). The area under the curve (AUC) was 0.617 at 1 year, at 2 years it reached 0.622, and at 3 years it reached 0.659 (Figure 5(f)) and reached 0.617 at risk, 0.757 at stage, 0.681 at grade, 0.609 at age, and 0.522 at gender in the ROC analysis (Figure 5(g)).

### 3.4. The Independent Prognostic Evaluation of Five Genes

**Figure 6:**
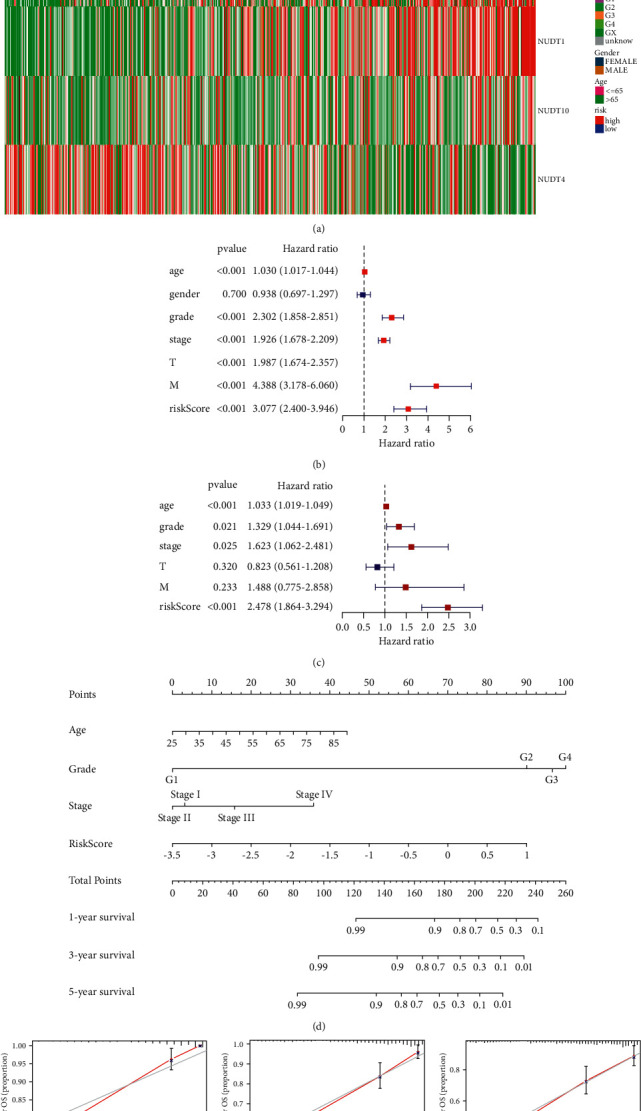
Construction of a new prognostic nomogram. (a) Heatmap and the clinicopathologic characters of the low- and high-risk groups. ^*∗*^*P* < 0.05, ^*∗∗*^*P* < 0.01, and ^*∗∗∗*^*P* < 0.001. (b) Univariate Cox regression analysis. (c)Multivariate Cox regression analysis. (d) Prognostic nomogram for predicting the survival of patients with ccRCC. The blue line represents a 95% confidence interval. The position of the square represents the hazard ratio. (e-g) Calibration curves of the nomogram for predicting survival at 1, 3, and 5 years. The nomogram prediction accuracy is higher if the actual curve is closer to the ideal curve. The red line represents the actual curve and the black line represents the ideal line.

We depicted to use a heatmap to illustrate the expression levels of the five selected m7G genes and clinical features in the two low-high groups, which suggested the significant differences in clinical features between the low and high groups (Figure 6(a)). Toward a better understanding of the predictive value of risk scores and other clinical features in ccRCC patients, the TCGA cohort was subjected to both univariate and multivariate Cox regression analyses (Figures 6(b) and 6(c)). We omitted the N-stage in our further analyses because most patients lacked clinical information on the N-stage. A prediction model was developed using four independent prognostic factors: age, risk score, grade, and stage. A nomogram predicting the OS probability over the course of 1, 3, and 5 years was estimated using the five m7G-related genes (Figure 6(d)) and assessed its predictive power (Figures 6(e) and 6(g)). The calibration curves proved that the nomogram had a positive effect on prognosis.

### 3.5. Functional Analyses in TCGA Cohort

**Figure 7:**
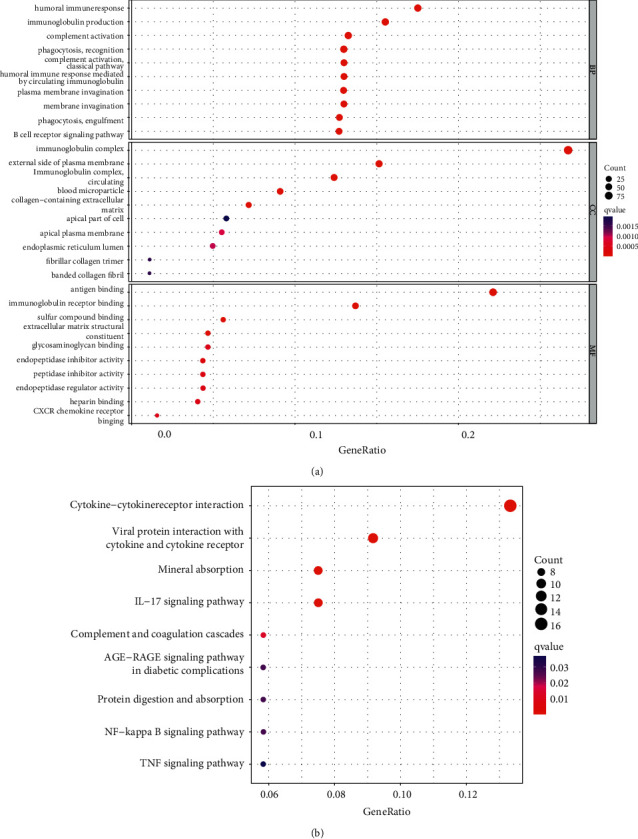
Results of GO and KEGG analysis of the TCGA cohort. (a) GO enrichment analysis revealed the biological processes and molecular functions involved in the differentially expressed genes. The GO enrichment analysis is divided into three parts, including cellular component (CC), molecule function (MF), and biological processes (BP). (b) KEGG analysis shows the signaling pathways involved in the differentially expressed genes.

To address the potential biological differences, we conducted GO and KEGG enrichment analyses on the DEGs between the different risk groups. The GO enrichment analysis of risk-related DEGs revealed significant correlations between humoral immune response, immunoglobulin complex, and antigen binding (Figure 7(a), *P* adjust <0.05). The KEGG pathway analysis demonstrated that DEGs had significantly enriched the following pathways: cytokine-cytokine receptor interaction, viral protein interaction with cytokine, and cytokine receptor and mineral absorption (Figure 7(b), *P* adjust <0.05).

### 3.6. Immune Infiltration ssGSEA in TCGA Cohort

**Figure 8:**
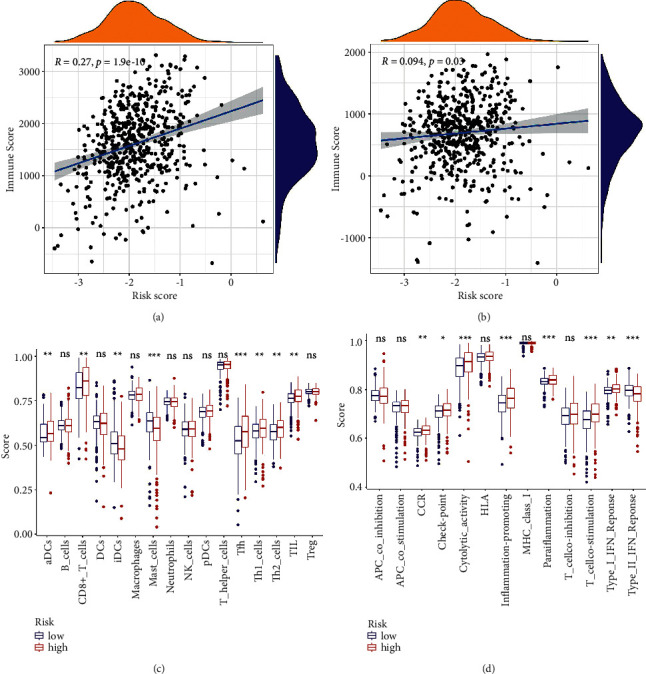
Results of ssGSEA immune infiltration in the TCGA cohort. The association between risk score and (a) immune score or (b) stromal score. (c) 16 immune cell ssGSEA scores between different risk groups. (d) 13 immune-related functional ssGSEA scores between different risk groups. Adjusted *P* values are: ns, not significant; ^*∗*^*P* < 0.05, ^*∗∗*^*P* < 0.01, and ^*∗∗∗*^*P* < 0.001.

The TCGA cohort was evaluated by ESTIMATE, which manifested a positive correlation between risk scores, immune scores (*P* < 0.001; Figure 8(a)), and stromal scores (*P*=0.03; Figure 8(b)). On six of the 16 immune cells, the high-risk group demonstrated significantly higher infiltration of activated dendritic cells (aDCs), CD8+ T cells, helper T cells 2 (Th2 cells), helper T cells 1 (Th1 cells), follicular helper T cells (Tfh), and tumor-infiltrating lymphocyte (TIL). In contrast, iDCs and mast cells showed the opposite pattern (*P* < 0.05, Figure 8(c)). In the immunopathways analysis, seven pathways were positively associated with risk scores, such as CCR, check-point, cytolytic-activity, inflammation-promoting, parainflammation, T cell co-stimulation, and type I IFN response. In comparison, the type II IFN response had a negative effect (*P* < 0.05, Figure 8(d)). The signature significantly correlates with immune infiltration, based on our findings.

### 3.7. Expressions of MRGs Correlated with Therapeutic Response

**Figure 9:**
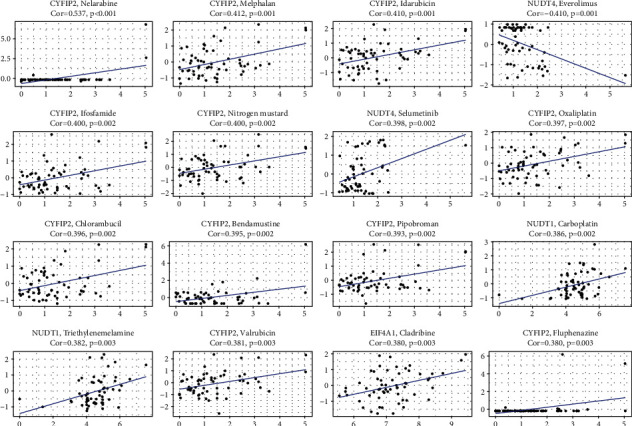
Correlation of m7G-related genes expressions with drug response. The relationship between drug sensitivity and CYFIP2, EIF4A1, NUDT1, NUDT1, and NUDT10 expression.

We incorporated the data on cancer cell expression and the effectiveness of FDA-approved drugs in our study in order to investigate the clinical applications of the m7G-related gene signature. CYFIP2-overexpressed cancer cells were demonstrated to be more sensitive to the following drugs: nelarabine, melphalan, idarubicin, and so on. The elevated NUDT4 expression levels in cancer cells increased the sensitivity to selumetinib, but drug resistance to everolimus was correlated with them. Overexpression of NUDT1 in cancer cells makes them more susceptible to carboplatin and triethylenemelamine, and cancer cells expressing higher levels of EIF4A1 were more sensitive to cladribine (Figure 9).

## 4. Discussion

Clear cell renal cell carcinoma is a fatal adult renal cancer [22], with a higher risk of recurrence and metastasis [23]. m7G and m6A are both common types of internal RNA modifications. Interestingly, numerous studies have revealed that m6A RNA methylation regulators are connected with the development and progression of cancer in humans [24], such as bladder cancer [25], hepatocellular carcinoma [26], and breast cancer [27]. However, there are few similar studies on m7G. Therefore, this study explored the impact of m7G RNA methylation regulators on ccRCC and constructed an m7G-related signature and nomogram for prognostic prediction of ccRCC.

First, we filtered out the differentially expressed m7G-related genes with prognostic values. Then we constructed the signature of five m7G-related genes, including CYFIP2, EIF4A1, NUDT1, NUDT10, and NUDT4. The expression of CYFIP2 and NUDT4 was downregulated, whereas EIF4A1, NUDT1, and NUDT10 expressions were upregulated in the ccRCC high-risk group. The cytoplasmic FMR1-interacting protein (CYFIP) family was initially recognized as a protein associated with brain diseases [28]. Recent research demonstrated that Cyfip 2 might play a critical role and have potential functions in cancers, such as gastric cancer [29] and colorectal adenocarcinoma [30]. Previous studies indicated CYFIP2 was downregulated in ccRCC patients due to its DNA methylation, and it was involved in the metabolic reprogramming, the EMT pathway, and immune infiltration processes in ccRCC by its DNA methylation [31]. The eukaryotic initiation factor 4A (eIF4A) family plays a vital role in many cancers [32–34]. In human cancers like gastric cancers and breast cancers, ZBP1 expression is increased, which also correlates with poor prognoses [35, 36]. EIF4A1 is a necessary component of EIF4F, which was considered a direct connection between essential steps in cancer development and translation initiation [37]. Moreover, accumulating studies have linked EIF4A1 to malignant phenotypes of tumor cells and tumor-specific survival [38, 39]. NUDT1, NUDT4, and NUDT10 belong to the nudix hydroxylase (NUDT) family. The expression of NUDT1 and NUDT10 is increased and the expression of NUDT4 is decreased in ccRCC [40]. Up to now, the association between the NUDT family and tumorigenesis has been unclear, and studies about their role in ccRCC are rare [41–43]. Previous studies indicated that NUDT1 might provide diagnostic and prognostic value for KIRC [44]. A study revealed that NUDT1 is activated by HIF2*α*transcription, thereby inhibiting oxidative stress and promoting the progression of ccRCC [45]. The roles of NUDT4 and NUDT10 remain ambiguous in ccRCC tumor progression and metastasis.

An analysis using Cox regression displayed that risk score, age, stage, and grade could act as the independent predictive factors for patients with ccRCC. These independent predictive factors were used to build a nomogram to improve the ability to predict the prognosis for ccRCC. The calibration curves demonstrated that the nomogram was fairly accurate in predicting the actual OS.

After performing GO, KEGG, and immune infiltration analyses on differential risk DEG genes, many biological pathways and functions were identified as contributing to immunity. It might be reasonable to conclude that m7G modification is closely related to tumor immunity. Lines of evidence demonstrate that the progression of ccRCC is associated with the presence of different types of immune cells and immune functions [46]. It has been proven that RNA methylation contributes to tumor immunity [47]. But the relationship between m7G modification and tumor immunity is still developing, and a deeper exploration of the mechanism is needed.

To facilitate the clinical transformation of the five genes model, we further identified FDA-approved sensitive drugs that express high levels of CYFIP2, EIF4A1, NUDT1, NUDT4, and NUDT10 in multiple cancer cell types. A high expression of CYFIP2 is related to cancer cells' sensitivity to nelarabine, Melphalan, and so on. Nelarabine is a process for obtaining the deoxyguanosine analog 9-*β*-Darabinofuranosylguanine(Ara-G) [48, 49]. Nelarabine has preferential cytotoxicity to T lymphoblastic cells through an accumulation of Ara-GTP, thereby inhibiting ribonucleotide reductase and subsequent DNA synthesis [50, 51]. According to the score comparisons of immune cells and immune function, we observed that high-risk patients possessed higher scores in helper T cells 2 (Th2 cells), CD8+ T cells, follicular helper T cells (Tfh), helper T cells 1 (Th1 cells), and T cell co-stimulation, suggesting a potential therapeutic agent in ccRCC with nelarabine. However, no literature has been found on the treatment of kidney cancer by nelarabine. Melphalan plus fludarabine is a feasible and effective RIC regimen for allogeneic SCT in metastatic RCC [52]. Increasing NUDT4 expression has been related to the resistance of tumor cells to everolimus. Nevertheless, it has been associated with increased sensitivity to selumetinib. Everolimus, a mammalian target of rapamycin (mTOR) inhibitor, is the standard second or third line therapy in patients with ccRCC [53]. SELUMETINIB inhibits MEK1/2, a mitogen-activated protein kinase, which can abrogate resistance, leading to improved antitumor efficacy in renal cell carcinoma [54]. NUDT1 expression was positively correlated with carboplatin and triethylenemelamine sensitivity in cancer cells. The increased expression of EIF4A1 was connected with higher sensitivity to cladribine in cancer cells. Carboplatin, triethylenemelamine, and cladribine are some of the most widely used cancer chemotherapeutics, but ccRCC is not sensitive to chemotherapy [55].

Currently, a large amount of literature on gene markers predicting the prognosis of ccRCC patients can be found. Some studies have constructed models without internal or external validation [56, 57] or only constructed prediction models without nomogram diagrams [57, 58]. Wenwei Chen and colleagues included age, sex, grade, and stage in univariate regression analysis but did not include TNM clinical indicators [59]. Zhao et al. constructed a nomograph diagram, including age, grade, stage, and risk score. Risk-score is classified as low and high rather than specific numerical values [56]. Compared with other gene predictive models of ccRCC patients, our research makes up for these deficiencies and has specific innovations and advantages. First of all, it is undeniable that our study is the first comprehensive study of m7G-related mRNA in ccRCC. In addition, the nomogram diagrams contain risk scores and clinical indicators that can further effectively predict the outcome of patients with ccRCC. Furthermore, Zhao et al. only evaluated the accuracy of the nomogram diagram, but not the accuracy of the gene-related model [56]. However, we evaluated the accuracy of the model using ROC. The closer the AUC is to 1.0, the higher the authenticity of the predictive model. Wang et al. also estimated the model's accuracy using ROC, and the risk AUC was 0.704 [60]. Our study demonstrates that the risk AUC is 0.748 in the TCGA cohort and the risk AUC is 0.811 in the E-MTAB-1980 cohort. It shows that our model is more accurate and effective. This study still has some flaws in its process and methodology. Firstly, the ccRCC cohort is relatively small, and the samples lack complete clinical information. Secondly, how those m7G-related genes interact with each other and which pathway is involved in ccRCC needs further study. Finally, the molecular mechanism of m7G-related genes in ccRCC remains to be conducted both in internal and external experiments.

## 5. Conclusion

Collectively, we constructed five m7G-related gene predictive models, which performed well in predicting survival prognosis, immune microenvironment, and sensitivity to drugs in ccRCC. Then, a prognostic nomogram for survival prediction based on five m7G-related genes could improve survival estimates for ccRCC patients. However, the potential mechanism of m7G-related genes in ccRCC remains unclear and needs further exploration.

## Figures and Tables

**Table 1:** Basic clinical characteristics of patients in the TCGA cohort and the E-MTAB-1980 Cohort.

	TCGA cohort (537)	E-MTAB 1980 cohort (101)
Age (years)		
≤65	352	57
>65	185	44
Gender		
Female	191	24
Male	346	77
Grade		
G1	14	13
G2	230	59
G3	207	22
G4	78	5
Gx	5	
Unknown	3	2
Stages		NA
I	269	
II	57	
III	125	
IV	83	
Unknown	3	
T Stage		
T1	275	68
T2	69	11
T3	182	21
T4	11	1
N Stage		
No	240	94
Not no	17	7
Nx	280	
M Stage		
M0	426	89
M1	79	12
Mx	30	
Unknown	2	

## Data Availability

All datasets are publicly available, and we can find all datasets here: https://portal.gdc.cancer.gov/and https://www.ebi.ac.uk/arrayexpress.
